# Missed opportunity for standardized diagnosis and treatment among adult Tuberculosis patients in hospitals involved in Public-Private Mix for Directly Observed Treatment Short-Course strategy in Indonesia: a cross-sectional study

**DOI:** 10.1186/1472-6963-10-113

**Published:** 2010-05-07

**Authors:** Ari Probandari, Lars Lindholm, Hans Stenlund, Adi Utarini, Anna-Karin Hurtig

**Affiliations:** 1 Department of Public Health, Faculty of Medicine, Universitas Sebelas Maret, Jl. Ir. Sutami 36A, Surakarta 57126, Indonesia; 2 Epidemiology and Global Health, Public Health and Clinical Medicine, Umeå University, Umeå, Sweden; 3 Department of Public Health, Faculty of Medicine, Universitas Gadjah Mada, Yogyakarta, Indonesia; 4 Swedish Research School for Global Health, Umeå, Sweden

## Abstract

**Background:**

The engagement of hospitals in Public-Private Mix (PPM) for Directly Observed Treatment Short-Course (DOTS) strategy has increased rapidly internationally - including in Indonesia. In view of the rapid global scaling-up of hospital engagement, we aimed to estimate the proportion of outpatient adult Tuberculosis patients who received standardized diagnosis and treatment at outpatients units of hospitals involved in the PPM-DOTS strategy.

**Methods:**

A cross-sectional study using morbidity reports for outpatients, laboratory registers and Tuberculosis patient registers from 1 January 2005 to 31 December 2005. By quota sampling, 62 hospitals were selected. Post-stratification analysis was conducted to estimate the proportion of Tuberculosis cases receiving standardized management according to the DOTS strategy.

**Result:**

Nineteen to 53% of Tuberculosis cases and 4-18% of sputum smear positive Tuberculosis cases in hospitals that participated in the PPM-DOTS strategy were not treated with standardized diagnosis and treatment as in DOTS.

**Conclusion:**

This study found that a substantial proportion of TB patients cared for at PPM-DOTS hospitals are not managed under the DOTS strategy. This represents a missed opportunity for standardized diagnoses and treatment. A combination of strong individual commitment of health professionals, organizational supports, leadership, and relevant policy in hospital and National Tuberculosis Programme may be required to strengthen DOTS implementation in hospitals.

## Background

The World Health Organization (WHO) has promoted the Directly Observed Treatment Short-Course (DOTS) strategy at the international level since the mid-1990s, and it has proved a cost-effective strategy to combat Tuberculosis (TB) [1-3]. DOTS strategy consists of five strategic pillars:

• Political commitment,

• Case detection by quality-assured sputum microscopy,

• Standardized short-course chemotherapy under direct observation of treatment,

• Quality-assured drugs,

• Recording and reporting system [4].

However, the implementation of DOTS strategy by public health facilities is insufficient to ensure the notification of all TB cases in the community as well as to provide adequate treatment and prevent further transmission [5,6].

Tuberculosis (TB) patients can receive care from a wide array of services, such as community health centres, general practitioners, traditional healers, chest clinics, and hospitals [7-11]. These facilities, however, do not necessarily implement the internationally-recommended DOTS strategy nor link to the National TB Programme. Evidence shows that without proper linkage to National TB Programme, these facilities are in fact providing poor quality diagnoses and treatment [12-15]. The need to engage different care providers in providing TB services is therefore urgent and the Public-Private Mix (PPM) for the DOTS initiative by WHO has been launched in response to this challenge [10,16].

Hospitals in particular play a major role as a source of TB treatment in many high-burden countries [11,17,18]; thus hospitals have been identified as priority targets for PPM-DOTS initiatives. Public-general and medical-college hospitals are the two types of health care providers most engaged in PPM-DOTS schemes [19]. Improved case detection and treatment outcome has been noted in several countries as the result of involving hospitals in PPM-DOTS strategy [19-22]. This favourable outcome has led to rapid scaling-up of hospital involvement. The number of high-burden countries adopting the hospital PPM-DOTS approach at the national level increased rapidly from 4 to 14 countries during 2005-2007 [19]; Indonesia is no exception, with an increase from 31% in 2005 (two years after the scaling up) to 37% of hospitals involved in PPM-DOTS by early 2007 (Unpublished data from Ministry of Health Republic of Indonesia).

Several publications have raised concerns regarding the quality of DOTS strategy implementation in hospitals [17,18,23-25]. Poor compliance with diagnostic and treatment guidelines, and the increase of Multi-Drug-Resistant-TB, further raises concerns about quality [18,24]. In view of the rapid global scaling-up of hospital engagement, we aimed to analyse the access to DOTS based services in hospitals already involved in PPM-DOTS strategy in Indonesia by determining the proportion of outpatient adult TB patients who actually received standardized diagnosis and treatment.

## Methods

### Study design

This was a cross-sectional study that was part of a larger research entitled: Assessment of the implementation of DOTS strategy in hospitals in six provinces on Java Island, Indonesia. The study was conducted from August 2006 to July 2007, with a pilot-study organized in three hospitals located in two provinces (Central Java and Yogyakarta).

**Figure 1:**
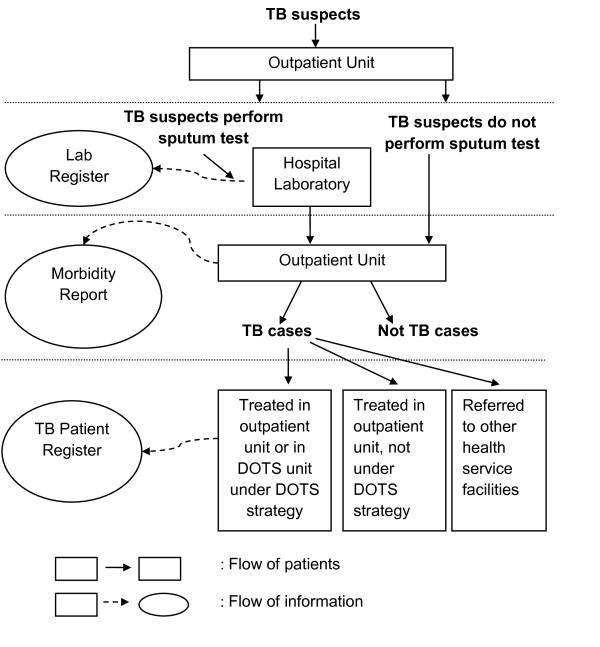
**Flow of patients and information among Tuberculosis cases in PPM-DOTS hospitals**.

Figure 1 describes the flow of patients and information about TB patients in hospitals. Depending on the main symptoms, TB suspects may have different entrances when using the outpatient service. They may visit general outpatient service or more specialized outpatient units (such as pulmonary, internal medicine, neurology, and surgery) prior to visiting a specially designated DOTS unit. To confirm the diagnosis, ideally a sputum smear examination should first be carried out and the results recorded in the laboratory register [26]. A certain proportion of TB suspects may also have to undertake other diagnostic tests simultaneously, most commonly a chest X-ray [17,18,27]. After completing the diagnostic tests, TB suspects return to the outpatient unit they initially visited. The diagnosis is recorded in the medical record, and later sent to the medical record department for the purpose of coding using the International Classification of Diseases (ICD) system. TB diagnoses are coded as ICD X A.15-A.19. However, it has been observed that the majority of hospitals do not record the specific ICD code but merge into a group of ICD codes for Tuberculosis. Based on the medical records, the hospital produces quarterly morbidity reports, including TB.

For treatment of TB patients, there are three possible scenarios. First, not all TB patients are managed in an outpatient unit with proper diagnosis and close monitoring of treatment using standardized TB patient register as in the DOTS strategy. Secondly, TB patients are treated with DOTS strategy in the unit where the patients were originally diagnosed or in the hospital DOTS unit (if available). Finally, confirmed TB patients are referred to other DOTS facilities closer to the patients' homes (such as to a community health centre). Due to incomplete recording of TB referrals from hospitals to other DOTS facilities, this study used the assumption of the referral rate from a study in Yogyakarta [22]. The median referral rate among hospitals during 2003-2005 was 31.5% for all TB cases and 32.6% for sputum smear positive TB cases [22].

For those treated with DOTS strategy, the TB patient registers are then sent to the TB supervisor at the District Health Office and the information is further aggregated at the province and national levels.

### Data collection

Trained surveyors collected the morbidity reports, laboratory registers and TB patient registers from the hospitals. Data from registers were double-entered. In order to improve the validity of data, the trained surveyors contacted the medical record staff to guarantee the completeness of the data and to clarify any issues arising.

### Study population, sampling strategy and sample

The study population included hospitals participating in PPM-DOTS strategy based on 2006 data from the National TB Programme in Indonesia. It consisted of 72 public general hospitals, 70 private general hospitals, and 8 pulmonary hospitals in Java Island, Indonesia. Providers from all these hospitals had taken part in standardized DOTS training activities conducted by the National TB Programme.

The sampling for the larger study was carried out using quota sampling. The quota was determined by consideration of the proportion, type, and ownership of PPM-DOTS hospitals among Java Island provinces. Based on type and ownership, the hospitals were differentiated into public general hospitals, private general hospitals, and public pulmonary hospitals. For this paper, we included hospitals in the study population which had been involved in PPM-DOTS for at least two years prior to 2006, and which had both a TB recording and reporting system and an outpatient-morbidity report system in place. Sixty-two hospitals met the inclusion criteria.

### Analyses

The analyses included, firstly, demographic characteristics of TB cases for each hospital group and evaluation of the differences by Kruskall Wallis tests and, secondly, post-stratification analysis to estimate the proportion of TB cases not treated under a DOTS unit. Post-stratification analysis estimated the weighted cumulative number of TB cases in the morbidity report and patient register as well as cases who were sputum smear-positive in the laboratory and TB registers. To calculate the post-stratification weight, the number of hospitals in the study population was divided by the number of sampled hospitals. The weighted cumulative number of TB cases was then estimated by multiplying the post-stratification weight by the cumulative number of estimated TB cases in the sample.

### Ethics

Approval for the study's ethics was received from Universitas Gadjah Mada, Indonesia. Permission for accessing the hospital registers was obtained from the local governments and hospitals. Patient confidentiality was assured during data analyses and presentation of study findings. The findings were shared with the hospitals, District-Provincial Health Offices and the National TB Programme.

## Results

The numbers of hospitals sampled were: 31 public general hospitals, 29 private general hospitals, and 2 pulmonary hospitals. All included hospitals have access to the National TB Programme guidelines and manual. Nevertheless, this does not mean that the hospitals have already integrated the National TB Programme guidelines into their standard operating procedure for TB patients seeking care at the hospital (Table 1). Not all hospitals have a formal Memorandum of Understanding for the implementation of the DOTS strategy.

**Table 1:** Characteristics of hospitals in the sample.

Characteristic	Public general hospitals (n = 31)		Private general hospitals (n = 29)		Public pulmonary hospitals (n = 2)	
	
	n	%	n	%	n	%
**Outpatient visits per year**						
<12,000	5	16.2	12	41.4	0	0
12,000-120,000	22	71.0	15	51.7	2	100
>120,000	4	12.9	2	6.9	0	0
Had NTP* guidelines and manual	31	100	29	100	2	100
**Had Standard of Operating Procedure for adult TB patients**	23	74.2	16	55.2	1	50
Had MoU**	12	38.7	13	44.8	0	0

*NTP = the National Tuberculosis Program; ** MoU = document of Memorandum of Understanding with the National Tuberculosis Programme.

The number of TB cases from the morbidity reports, laboratory registers and TB patient registers had a skewed distribution. In general, public pulmonary hospitals registered a higher number of TB cases per hospital compared to general hospitals. The medians were 712 in pulmonary hospitals, 247 in public general hospitals and 102 in private general hospitals (p = 0.03) (Table 2). Furthermore, the total number of TB cases (n = 349) registered in pulmonary hospital DOTS units was higher than in public (n = 52) or private (n = 19) general hospitals (p = 0.01). Similarly, the number of sputum smear positive TB cases identified by the pulmonary hospital laboratories (n = 198) was greater than in public (n = 43) or private (n = 17) general hospitals (p = 0.001). A similar pattern was found for the number of sputum smear positive TB cases undergoing treatment at DOTS units (p = 0.004).

**Table 2:** Number of Tuberculosis cases: comparison of morbidity reports, laboratory registers and TB patient registers at general hospitals and pulmonary hospitals, 2005.

	Public general hospital	Private general hospital	Public pulmonary hospital	All hospitals
**Number of hospitals**	31	29	2	62
TB* cases in morbidity report (n)**				
Median (min-max)	247 (5-1,601)	102 (5-1,584)	712 (631-793)	211 (5-1,601)
Cumulative	11,223	6,843	1,424	19,490
**TB cases in TB patient register (n)**				
Median (min-max)	52 (1-169)	19 (2-170)	349 (264-434)	37.5 (1-434)
Cumulative	1,710	1,140	698	3,522
SS (+)***TB cases in laboratory register (n)				
Median (min-max)	43 (0-296)	17 (0-115)	198 (157-239)	35 (0-296)
Cumulative	2,053	929	396	3,378
**SS (+) TB cases in TB patient register (n)**				
Median (min-max)	25 (2-126)	9 (2-80)	117.5 (113-122)	17 (0-126)
Cumulative	1,011	587	235	1,833

* TB = tuberculosis; **n = number; ***SS (+) = sputum smear positive.

Results of the post-stratification analysis showed the discrepancy between the number of TB cases recorded in the TB patient register and those in the hospital morbidity report. After adjusting for the referral rate, the proportion of TB cases not recorded in TB patient register was 53% (in public general hospitals) and 52% (in private general hospitals). This proportion was larger in general hospitals than that in public pulmonary hospitals (i.e. 19.5%) (Table 3).

**Table 3:** Result of post-stratification analysis.

	Public general hospital	Private general hospital	Public pulmonary hospital	All hospitals
Hospitals in the study population (n)*	72	70	8	150
Hospitals in the sample (n)	31	29	2	62
Post-stratification weight	2.32	2.41	4.00	2.42
Weighted cumulative number of TB** cases in morbidity report (A)	26,066	16,518	5,696	47,153
Weighted cumulative number of TB cases in TB patient register (B)	3,972	2,752	2,792	8,521
Weighted cumulative number of SS (+)*** TB cases in laboratory register (C)	4,768	2,242	1,584	8,173
Weighted cumulative number of SS (+) TB cases in TB patient register (D)	2,348	1,417	940	4,435
Adjusted gap of cumulative number TB cases between TB patient register and morbidity report, applying referral rate of 31.5% [(100-(B/A))-31.5%]	53.3%	51.8%	19.5%	50.4%
Adjusted gap of cumulative no. SS (+) TB cases between TB patient register and Lab register, applying 32.6% of referral rate [(100-(D/C))-32.6%]	18.2%	4.2%	8.1%	13.1%

*n = number; **TB = tuberculosis; ***SS (+) = sputum smear positive.

After considering the referral rate, the gap between the number of sputum smear positive TB cases recorded in the laboratory register and those recorded in the TB patient register ranged from 4% to 18%. The highest proportion was in public general hospitals, followed by public pulmonary hospital and private general hospitals (18.2%, 8.1%, and 4.2% respectively) (Table 3).

## Discussion

The findings show sub-optimal access to the DOTS strategy among TB patients in hospitals involved in the PPM-DOTS initiative. This inconsistent access to DOTS strategy is more prominent in public general hospital whereas the general and medical college hospitals are the most common type of hospitals involved in PPM-DOTS [19]. A similar phenomenon was also reported in a study by Loveday *et al*. (2008), which found that 58% of smear positive TB cases did not access standardized National TB Programme treatment in the hospitals [18]; this could be due to a lack of cooperation in the application of diagnosis and treatment based on DOTS strategy for all TB patients [15,28], perceived complexity of DOTS based diagnosis and treatment [29], and perceived low quality of services due to provision of free TB drugs [29].

Taking into account the reliance on secondary data in this study, the findings still raise the issue of missed opportunity for PPM-DOTS hospitals to deliver quality diagnosis and treatment for all TB suspects. This could lead to misdiagnosis of TB patients and, consequently, improper treatment of TB patients. With the present, and alarming, problem of multiple-drug-resistant TB [18,24], low quality of TB case management in hospitals implementing PPM-DOTS strategy certainly requires urgent attention. Therefore, we argue that a better balance is required between the expansion of PPM-DOTS strategy to new hospitals, and improvement of quality DOTS strategy implementation in existing hospitals.

Since services for TB patients can be delivered at several outpatient units in collaboration with other units (DOTS units, laboratories, medical records units, etc), the findings also reflected the complexity of internal linkages between those different micro systems involved in delivering TB care in hospitals. Several factors may contribute to the weakening of internal linkages, i.e. from micro system to organization level, up to the policy at the national level. At the micro system level, individual commitment of health professionals [25], as well as teamwork, information, performance and improvement, and clinical leadership [30] are key factors. Subsequently, hospital and National Tuberculosis Programme policies that help to support and strengthen those factors (in order to improve quality) are critical.

Different mechanisms exist to enhance the quality of DOTS strategy implementation in hospitals. At the micro system level, launching of the International Standard for TB Care can be an initial bridge for improving the commitment of health professionals involved in delivering TB care [31,32]. Endorsement from professional organizations and operational support to ensure implementation of the International Standard for TB Care among specialists are required [33]. Staff incentives are important to focus the staff on providing high quality services to patients [30]. However, there is limited evidence of the types of incentives that are effective in the context of PPM-TB control [34,35]. At the hospital level, Siddiqi *et al*. (2008) implemented TB clinical audit as a process measurement for improving clinical TB care [36]. Experience in the Philippines suggests that national level regulations such as accreditation and certification of PPM-DOTS hospitals can also be effective in improving the quality of TB services [34]. Finally, existing mechanisms in TB control management, i.e. internal and external supervision, should also be strengthened to improve practices in the context of PPM-DOTS hospitals.

This study is limited to measure, not explain, the phenomenon of missed opportunities on TB diagnosis and treatment in PPM-DOTS hospitals. The assumed referral rate for sputum smear positive TB cases may be considered too high for Java Island and other areas in Indonesia because the rates were calculated under a closely monitored pilot project. If this is the case, our results in fact underestimate the proportion of cases not administered under DOTS strategy.

## Conclusions

This study found that a substantial proportion of TB patients cared for at PPM-DOTS hospitals are not managed under the DOTS strategy. This represents a missed opportunity for standardized diagnoses and treatment. A combination of strong individual commitment of health professionals, organizational supports, leadership, and relevant policy in hospital and National Tuberculosis Programme may be required to strengthen DOTS implementation in hospitals.

## Authors' contributions

AP was responsible for developing the research idea, designing the study, executing the data collection, analysis and interpretation of the results, as well as for the writing of the manuscript. LL and HS contributed to the interpretation of the data, and revision of the manuscript. AU participated in the study design, execution of the data collection, analysis and interpretation of the data, as well as in substantially revising the manuscript. AK gave intellectual inputs to the study design, analysis and interpretation of data and performed the critical revision of the manuscript. AU and AK made equal contribution to the study. All authors read and approved the final manuscript.

## Competing interests

The authors declare that they have no competing interests.

## Pre-publication history

The pre-publication history for this paper can be accessed here:

http://www.biomedcentral.com/1472-6963/10/113/prepub
